# The risks of social distancing for older adults: a call to balance

**DOI:** 10.1017/S1041610220001350

**Published:** 2020-06-24

**Authors:** Myrra Vernooij-Dassen, Frans Verhey, Maria Lapid

**Affiliations:** 1 Scientific Center for Quality of Healthcare, Radboud University Medical Center, Nijmegen, The Netherlands; 2 Department of Psychiatry and Neuropsychology, Maastricht University Medical Center, Maastricht, The Netherlands; 3 Department of Psychiatry and Psychology, Mayo Clinic, Rochester, MN, USA

## Introduction

It is argued that the psychosocial implications of the COVID-19 pandemic exacerbate and often supersede its direct medical impact (Ayalon, 2020). This might be due to social distancing as the key strategy to effectively fight the spread of the COVID-19 infection. While social distancing can protect and save lives, its deleterious effects on older individuals need also to be recognized and minimized in order to preserve their quality of life to the extent possible. Social distancing deprives older adults from direct interaction with their social environment and thereby disturbs the potential of social health to preserve their quality of life. The social capacities of older adults and the response of their social environment are powerful means to adapt to challenging situation such as a pandemic by social interactions stimulating mental health and cognitive functioning. However, being disconnected from loved ones and people giving pleasure and meaning to life constitutes an additional risk and makes older adults more vulnerable to loneliness and to deterioration of mental and cognitive functioning.

In this commentary, we review the impact of social distancing on mental and social health and on cognitive functioning and describe practical strategies to counteract the adverse effects of social distancing on older individuals.

## Social distancing

Social distancing or “physical distancing” is defined globally as maintaining at least 6 feet of distance from other people, avoiding crowds or large gatherings, and staying at home. Social distancing is designed to reduce interaction between people in a broader community in which individuals may be infectious, but have not yet been identified, hence not yet isolated. Social distancing may reduce transmission of respiratory droplets (Wilder-Smith and Freedman, 2020). The most vulnerable persons in the COVID-19 outbreak are older adults (>70 years of age).

## Adverse impact of social distancing in older individuals

Social distancing affects society and social life by changing cultural habits and increases risk for impairments in social and mental health and cognitive functioning.

### Social health

Social distancing challenges social health. Social health is a new paradigm bridging biomedical and social sciences by emphasizing the role of social interaction in managing health, formulated as the ability to adapt and self-manage. Social health reflects the competencies of the individual to participate in social interaction and the influence of the social environment on the individual’s balance of capacities and limitations (Huber *et al.*, 2011; Vernooij-Dassen *et al.*, 2019). Social health ranges from a flourishing social life to loneliness. It is a dynamic process in which the individual is the conductor. Conducting complex social processes seems to be supported by wisdom. Wisdom might affect the quality of social relationships positively: loneliness has been found to be correlated strongly and inversely with wisdom (Lee *et al.*, 2019). In contrast to public opinion, loneliness severity and age had a nonlinear relationship with increased loneliness in the late 20s, mid 50s, and late 80s (Lee *et al.*, 2019). Social distancing restricts individuals reaching out to their social environment and vice versa. It also deprives older adults from meetings that allow them to fulfill spiritual needs and meet family and friends. For instance, for many Filipinos going to church on Sunday followed by family gatherings is a tradition they look forward to, but now adds to a sense of sadness (Buenaventura *et al.*, 2020). Thus, social distancing deprives people of the many valuable assets of social health: face-to-face interactions characterized by sharing emotions such as pleasure as well as physical closeness.

Face-to-face interactions have been replaced by e-communications. Technology is a great communication facilitator. But older adults need more than virtual contacts. When older adults are facing the challenges of social isolation, they are particularly vulnerable to rapid decline (Steinman *et al.*, 2020). This is especially evident in long-term care facilities.

In long-term care facilities, there is not only a high virus outbreak but also an outbreak of loneliness. Dying alone, due to the social distancing measures, is major fear and the last thing we want. The tragedy is that the very measure designed to protect older adults (namely, social isolation) endangers their quality of life and even their quality of dying.

### Mental health

Social distancing in its extreme form by quarantine and social isolation during previous periods of severe coronavirus outbreaks has been found to be associated with mental health problems such as depression, anxiety, and stress (Rohr *et al.*, 2020). More than 50% of Chinese people participating in a COVID-19 study (*n* = 1210) rated negative psychological impact of the outbreak, with 28.8% of the participants reporting moderate to severe anxiety (Wang *et al.*, 2020).

### Cognitive functioning

Quarantine had negative associations with cognitive functioning (Rohr *et al.*, 2020). These risks need more attention especially since social distancing may be mandatory for a longer period in some cases.

There is a striking consistency between these results and those of dementia research. A lack of social interaction is associated with incident dementia (Kuiper *et al.*, 2015). Conversely, epidemiological data indicate that a socially integrated lifestyle had a favorable influence on cognitive functioning (Bellou *et al.*, 2017) and could postpone the onset of dementia (Fratiglioni *et al.*, 2004). Hypothetically, social interactions may trigger reactions which might require the use of preexisting cognitive processes or activating compensatory approaches (Fratiglioni *et al.*, 2004; Vernooij-Dassen *et al.*, 2019).

How to explain this potential of the brain to improve cognitive functioning? It is in the plasticity of the brain. The plasticity of the brain allows activating preexisting cognitive processes or activating compensatory approaches: the cognitive reserve (Fratiglioni *et al.*, 2004). This is a very promising angle in dementia research.

But what is the working mechanism in relation to social health?

We hypothesize that social health can act as the driver for accessing cognitive reserve through active utilization of social resources (Vernooij-Dassen *et al.*, 2019). It reflects hope on how to prevent dementia and to mitigate its consequences. Social interactions for humans are like water for plants.

Social interactions during the COVID-19 crisis are embedded within intergenerational solidarity. With an impressive intergenerational solidarity, the first outbreak wave has been managed. But intergenerational solidarity is shifting. Younger generations want to resume normal life and suggestions are being made about excluding those aged 60 plus from societal activities in order to regain normality. Ageism is playing a role. Almost one quarter of analyzed tweets during the COVID-19 outbreak had ageist content (Jimenez-Sotomayor *et al.*, 2020).

Political decision makers are confronted with these tendencies. In this new unlocking phase of the COVID-19 crisis, it is crucial to reduce the negative consequences by limiting social distancing as much as possible. It is the balance between protection and its risks that counts. This is a call to translate this balance into policies that do not challenge intergenerational solidarity and lead to ageism. Therefore, the needs of all generations should be considered without excluding the older generation.

## Strategies to mitigate the impact of social distancing

There are a number of strategies that geriatric providers, policy makers, community groups, and older individuals themselves can do to lessen the negative impact of social distancing on social and mental health and cognitive functioning of older individuals.

- To optimize social health and ease social isolation despite social distancing measures: increase social contacts; engage with a person really close to you, especially when you are living alone; take personal responsibility for managing feelings of loneliness (Kharicha *et al.*, 2020); and replace touching by verbal expression of affection.
- To optimize mental health: prevent depression by reaching out especially when you do not consider yourself in need of it. Mental health systems should prepare for psychological first aid to meet patients’ needs.
- To optimize cognitive functioning: make chats during the day about what ever topic (Holt-Lunstad *et al.*, 2010) and organize leisure activities with other persons.
- Psychosocial interventions for very frail persons: visit people with dementia as much as possible (www.alzheimer-europe.org); use all kinds of communication tools including telephone and video communication (www.interdem.org blog Chattat); provide palliative care and use advance care planning to consider needs and wishes (Tilburgs *et al.*, 2019).

## Conclusions

Social distancing is meant to save the lives of vulnerable older adults, but it comes with high costs. The strategic response to the COVID-19 crisis should not only aim to save lives, develop effective treatments, and revive the economy but also protect individuals and societies from the social, mental, and cognitive adverse effects of social distancing. Social inclusion is feasible and rewarding for both vulnerable persons and society.
